# Clinico-pathological and gene features of 15 nemaline myopathy patients from a single Chinese neuromuscular center

**DOI:** 10.1007/s13760-023-02333-8

**Published:** 2023-07-31

**Authors:** Lv Haidong, Liu Yin, Chen Ping, Zheng Xianzhao, Qian Qi, Ma Xiaoli, Lv Zheng, Cui Wenhao, Zhou Yaguang, Qu Qianqian

**Affiliations:** 1 Department of Neurology, Jiaozuo People’s Hospital of Henan Province, Henan, 454002 Henan Province People’s Republic of China; 2 Department of Neurology, Renmin Hospital of Wuhan University, Wuhan, 430060 Hubei Province People’s Republic of China

**Keywords:** Nemaline myopathy, Genetics, Pathogenesis, Clinical features

## Abstract

**Background:**

Nemaline myopathy, the most common of the congenital myopathies, is caused by various genetic mutations. In this study, we attempted to investigate the clinical features, muscle pathology and genetic features of 15 patients with nemaline myopathy.

**Results:**

Among the 15 patients, there were 9 (60.00%) males and 6 (40.00%) females, and 9 (60.00%) of them came from three families respectively. The age of seeing a doctor ranged from 9 to 52 years old, the age of onset was from 5 to 23 years old, and the duration of disease ranged from 3 to 35 years. Ten out of the 15 patients had high arched palate and elongated face. Only one patient had mild respiratory muscle involvement and none had dysphagia. Muscle biopsies were performed in 9 out of the 15 patients. Pathologically, muscle fibers of different sizes, atrophic muscle fibers and compensatory hypertrophic fibers could be found, and occasionally degenerated and necrotic muscle fibers were observed. Different degrees of nemaline bodies aggregation could be seen in all 9 patients. The distribution of type I and type II muscle fibers were significantly abnormal in patients with nemaline myopathy caused by NEB gene, however, it was basically normal in patients with nemaline myopathy caused by TPM3 gene and ACTA1 gene. Electron microscopic analysis of 6 patients showed that nemaline bodies aggregated between myofibrils were found in 5(83.33%) cases, and most of them were located near the Z band, but no intranuclear rods were found. The gene analysis of 15 NM patients showed that three NM-related genes were harbored, including 11 (73.33%) patients with NEB, 3 (20.00%) patients with TPM3, and 1 (6.67%) patient with ACTA1, respectively. A total of 12 mutation sites were identified and included 10 (83.33%) mutations in exon and 2(16.67%) mutations in intron.

**Conclusions:**

The clinical phenotype of nemaline myopathy is highly heterogeneous. Muscle pathology shows that nemaline bodies aggregation is an important feature for the diagnosis of NM. NEB is the most frequent causative gene in this cohort. The splicing mutation, c.21522 + 3A > G may be the hotspot mutation of the NEB gene in Chinese NM patients.

## Background

Nemaline myopathy (NM) is one of the most common forms of congenital myopathy. NM is defined by the presence of rod-like structures in skeletal muscle fibers, which was first described by Shy and Conen in 1963 [1, 2]. The main clinical manifestations include hypotonia and muscle weakness, and respiratory muscles may be involved in severe cases. Some are accompanied by facial and bone deformities. Under the light microscope, dark blue rods can be found in fibers on skeletal muscle biopsy, which is the characteristic histologic feature of NM. Fourteen NM causative genes have been reported in the literature, including *NEB, ACTA1, TPM3, TPM2, TNNT1, TNNT3, CFL2, KBTBD13, KLHL40, KLHL41, LMOD3, MYO18B, MYPN, RYR3* [3–16], among which *NEB* gene is the most commonly reported gene [17]. Recently, it has been reported that rods can be found in myopathy caused by the *CAP2* gene [18]. Here, we systemically review the clinical, pathological and genetic characteristics of 15 patients with NM in our neuromuscular Center, and discuss the clinical phenotype of NM in Chinese population and the mutation characteristics of three common NM pathogenic genes, which are *NEB, ACTA1* and *TPM3*.

## Patients and methods

### Patients

A total of 15 patients were included in the retrospective study. From January 1997 to January 2021, muscle biopsies were performed on patients with suspected myopathy in our neuromuscular Center, and 15 patients were diagnosed with NM according to the characteristic pathological features. Detailed clinical data were collected from 15 patients, which included gender, age at onset, clinical course, initial symptom, distribution and grade of muscle weakness, dysmorphic features, creatine kinase (CK), Electromyography (EMG) results and family history. Written informed consent was obtained from each patient, and this study was approved by the Medical Ethics Committee of Jiaozuo People's Hospital.

### Pathological analysis

Open muscle biopsies were performed in 9 patients under local anesthesia in our cohort, which included quadriceps femoris muscle (n = 5), tibialis anterior muscle (n = 2) and gastrocnemius muscle (n = 2). A portion of the muscle specimens was fixed with liquid nitrogen and frozen into sections for routine histological and histochemical staining. The staining included hematoxylin and eosin (H&E), modified Gomori trichrome (MGT), Periodate Schiff reaction (PAS), oil red "O" (ORO), and adenosine triphosphatase (ATP) at varying pHs (pH = 4.2, 4.3, 10.3, 10.4) nicotinamide adenine dinucleotide tetrazolium reductase (NADH-TR). The stained slides were examined under the light microscope. The other portion of the muscle biopsy was fixed in 2.5% glutaraldehyde and osmium acid, dehydrated and embedded in plastic, and made ultrathin sections which were observed under the Transmission Electron Microscope (TEM).

### Molecular analysis

Genetic testing was performed on 15 patients with the informed consent of the patients and their families. Genomic DNA was extracted from peripheral blood samples (n = 12) or muscle tissues (n = 3) from all patients using standard procedures, to screen genome-wide exon single-gene genetic disease, which was performed at the Genetic Testing Center.

## Results

### Clinical features

Among the 15 patients, there were 9 (60.00%) males and 6 (40.00%) females, of whom 9 (60.00%) came from three families, respectively. The age of seeing a doctor ranged from 9 to 52 years old, with a mean age of 31.53 ± 10.94 years the age of onset was ranged from 5 to 23 years old, with a mean age of 13.73 ± 4.33 years. The duration of disease ranged from 3 to 35 years, with a mean disease course of 17.80 ± 9.49 years. All patients presented with weakness of lower limbs mainly manifested as difficulty in squatting and standing up and weakness in foot dorsiflexion. Neurological examination showed normal cranial nerves, muscle tone of both upper and lower limbs was normal or decreased and deep tendon reflexes was reduced. The proximal muscle strength was ranged from 3/5 to 5/5, and the distal was ranged from 4/5 to 5/5. The muscle tone of both lower limbs was reduced and the tendon reflex at knee and ankle was absented. The proximal muscle strength was ranged from 2/5 to 4/5 and the distal was ranged from 2/5 to 5/5. Ten out of the 15 patients had high arched palate and elongated face. Only one patient had mild respiratory muscle involvement. The creatine kinase was normal or slightly elevated in all patients. Details of the clinical manifestations of the 15 patients were shown in Tables 1, 2.

**Table 1:** Demographics, clinical characteristics of 15 patients

Patient	Gender	Age	Course of disease	Family history	Initial Symptom	Muscle Weakness Grading				Deformity	Creatinase(U/L)
						Upper limbs		Lower limbs			
1	M	10	5	No	Lower limbs weakness	Proximal V	Distal V	Proximal IV	Distal III	Pes cavus	132
2	M	20	11	No	Lower limbs weakness	Proximal V	Distal V	Proximal IV	Distal II	Pes cavus	76
3	M	27	9	No	Lower limbs weakness	Proximal IV	Distal V	Proximal IV	Distal IV	High arched palate, elongated face	198
4	F	52	35	Yes	Lower limbs weakness	Proximal IV	Distal IV	Proximal III	Distal IV	High arched palate, elongated face	64
5	M	49	33	Yes	Lower limbs weakness	Proximal V	Distal V	Proximal IV	Distal IV	High arched palate, elongated face	153
6	M	45	32	Yes	Lower limbs weakness	Proximal IV	Distal IV	Proximal IV	Distal IV	High arched palate, elongated face	137
7	M	34	15	No	Lower limbs weakness	Proximal V	Distal V	Proximal III	Distal IV	High arched palate, elongated face	89
8	M	34	11	No	Lower limbs weakness	Proximal III	Distal lV	Proximal III	Distal lV	Pes cavus	129
9	M	31	18	Yes	Lower limbs weakness	Proximal V	Distal lV	Proximal III	Distal V	High arched palate, elongated face, pes arcuatus	201
10	F	32	19	Yes	Lower limbs weakness	Proximal V	Distal V	Proximal III	Distal V	High arched palate, elongated face, pes arcuatus	131
11	F	28	13	Yes	Lower limbs weakness	Proximal V	Distal V	Proximal IV	Distal V	High arched palate, elongated face, pes arcuatus	117
12	F	30	20	Yes	Lower limbs weakness	Proximal V	Distal V	Proximal III	Distal III	High arched palate, elongated face	115
13	F	33	23	Yes	Lower limbs weakness	Proximal V	Distal V	Proximal IV	Distal IV	High arched palate, elongated face	146
14	F	32	20	Yes	Lower limbs weakness	Proximal V	Distal V	Proximal IV	Distal IV	High arched palate, elongated face	164
15	M	16	3	No	Lower limbs weakness	Proximal V	Distal V	Proximal IV	Distal I V	No	252

**Table 2:** Summary of genetic features characteristics of 15 patients

Patient	Gene Mutatio	Chromosome position	Inherited pattern	c.DNA variant	Exon/Intron	Amino acid variant	Variants pathogenicity
1	NEB	2	Sporadic	c.2549delA,; c.21522 + 3A > G	Ex27 In143	p.(Lys850fs); Splicing	Pathogenic
2	NEB	2	Sporadic	c.21065dupA; c.21522 + 3A > G	Ex140 In143	p.H7022Qfs*9; Splicing	Pathogenic/Pathogenic likel
3	NEB	2	Sporadic	c.4417C > T; c.21522 + 3A > G	Ex38 In143	p.R1473*; Splicing	Pathogenic/Pathogenic likely
4	NEB	2	AR	c.1623delT; c.21522 + 3A > G	Ex18 In143	p.(Asp542fs); Splicing	Pathogenic/Pathogenic likely
5	NEB	2	AR	c.1623delT,; c.21522 + 3A > G	Ex18 In143	p.(Asp542fs); Splicing	Pathogenic/Pathogenic likely
6	NEB	2	AR	c.1623delT; c.21522 + 3A > G	Ex18 In143	p.(Asp542fs); Splicing	Pathogenic/Pathogenic likely
7	NEB	2	Sporadic	c.3520G > A; c.21522 + 3A > G	Ex33 In143	p.A1174T; Splicing	VUS/Pathogenic likely
8	NEB	2	Sporadic	c.192G > A; c.20943G > A	Ex139 Ex5	p.(Val6981 =); p.(Lys64 =)	VUS/VUS
9	NEB	2	AR	c.17611C > T; c.21522 + 3A > G	Ex111 In143	p.(Gln5871*); Splicing	Pathogenic/Pathogenic likely
10	NEB	2	AR	c.17611C > T; c.21522 + 3A > G	Ex111 Ex143	p.(Gln5871*); Splicing	Pathogenic/Pathogenic likely
11	NEB	2	AR	c.17611C > T; c.21522 + 3A > G	Ex111 Ex143	p.(Gln5871*); Splicing	Pathogenic/Pathogenic likely
12	TPM3	1	AR	c.642 + 5G > A; c.*25G > A	In6 Ex10	Splice; utr-3	VUS/VUS
13	TPM3	1	AR	c.642 + 5G > A; c.*25G > A	In6 Ex10	Splice; utr-3	VUS/VUS
14	TPM3	1	AR	c.642 + 5G > A; c.*25G > A	In6 Ex10	Splice; utr-3	VUS/VUS
15	ACTA1	1	AD	c.745G > A	Ex5	p.V249I	VUS

### Pathological features

**Fig. 1:**
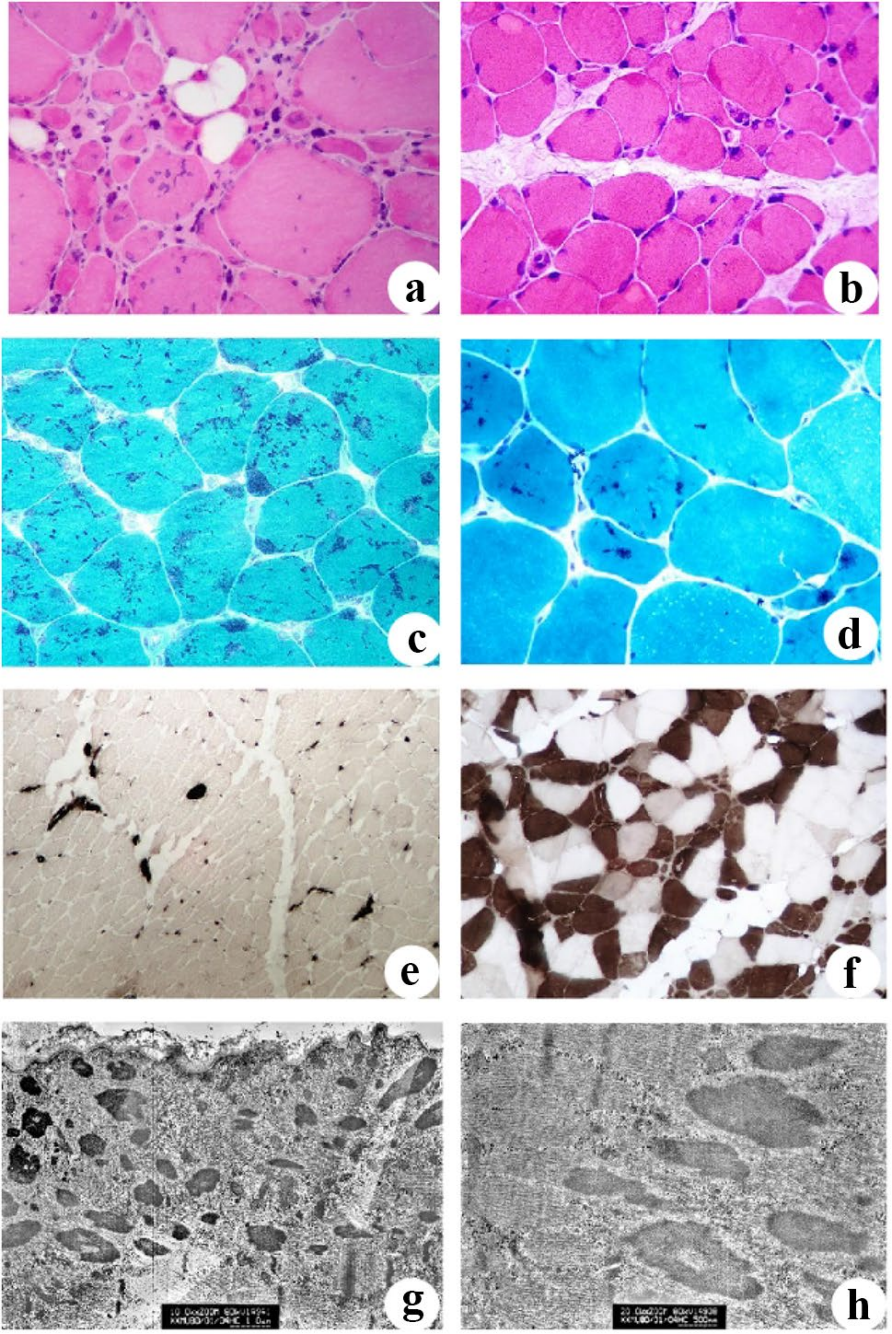
**a** HE staining showed that the muscle fibers were of different sizes, round or strip atrophic muscle fibers and compensatory hypertrophic muscle fibers (HE, × 400); **b** eosinophilic substance assembled in some muscle fibers, most of which were located under the sarcolemma (HE, × 400); **c**, **d** MGT staining showed that dark blue rod-shaped deposits (nemaline bodies) were observed in different muscle fibers (MGT, × 400); **e** ATP staining indicated that the distribution of typeI and typeII muscle fibers was abnormal, and type 1 fibers were significantly predominant, type II fibers were rare (ATPase10.3, × 200); **f** ATP staining revealed that type I and type II fibers were distributed in a mosaic pattern (ATPase10.3, × 200); **g**, **h** Electron microscopy showed many rods aggregated among subsarcolemmal and myofibril, and the rods were mostly located near Z line (EM lead-axis double staining, × 20,000)

**Fig. 2:**
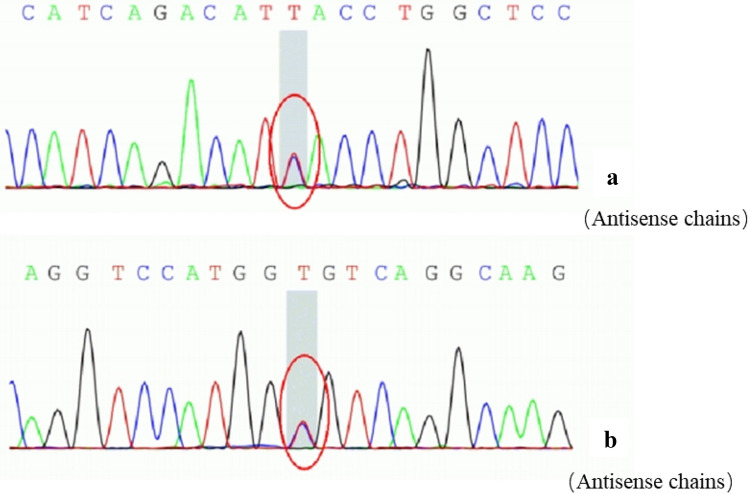
Sanger sequencing of NEB gene in case 7. **A** Heterozygous mutation c.21522 + 3A > G at intron 144 (red circle); **B** Heterozygous mutation c.3520G > A in exon 33 (red circle) (Color figure online)

Muscle biopsies were performed in 9 patients, and the muscle fiber sizes in 5 (55.56%) patients were significantly variable, while in 4 (44.44%) patients were slightly variable, and some fibers had nuclear inward migration (Fig. 1a). The eosinophilic substances assembled subsarcolemmal or in the center were observed in 4 (44.44%) patients (Fig. 1b). A few degenerated and necrotic fibers with a small amount of phagocyte infiltrating were occasionally observed in 3 (33.33%) patients. Dark stained rods were observed in muscle fibers of all 9 patients (Fig. 1c, d), and the involved myofibrillar structure was disarranged in 4 (44.44%) patients. ATPase staining indicated that the distribution of type I and type II fibers in patients with NM caused by *NEB* was abnormal, of which 5 (55.56%) patients were type I fiber predominance (Fig. 1e) and 2 (22.22%) patients were type II fiber predominance. The other 2 (22.22%) patients with NM caused by *TPM3* and *ACTA1* gene mutations respectively, had a normal checkerboard pattern of Type I and Type II fibers (Fig. 1f). Electron microscopic analysis showed that multiple focal myofibrillar structures disorganized, Z disc disappeared or disrupted, rods mostly located near the Z line accumulated in myofibril were found in 6 (66.67%) cases (Fig. 1g, h). No Intranuclear rods were found. Pathological changes in 9 patients with biopsies were shown in Table 3.

**Table 3:** Summary of pathological features of 15 nemaline myopathy patients

Patient	Skeletal muscle biopsy location	Muscle fiber atrophy/necrosis (H&E)	Eosinophilic aggregates (H&E)	Nuclear inward migration (H&E)	Proportion of fiber containing rods (MGT)	Site of aggregation of rods (MGT)	Myofibrillar network disorder (NADH)	Predominant fiber type (ATPase)	Position of rods under electron microscope	
									Intracellular/cytoplasmic	Intranuclear
1	Quadriceps femoris	±	+	–	50%	Central	–	II	+	–
2	Gastrocnemius	+ + / +	–	+	90%	Central and subsarcolemmal	+	I	+	–
3	Tibialis anterior	+ + / +	+	+	90%	Central and subsarcolemmal	+	I	+	–
4	Gastrocnemius	+ ±	+	+	70%	Central and subsarcolemmal	+	I	+	–
5	/	/	/	/	/	/	/	/	/	/
6	/	/	/	/	/	/	/	/	/	/
7	Tibialis anterior	±	+	+	90%	Central and subsarcolemmal	–	II	+	–
8	Quadriceps femoris	+ + / +	–	+	30%	Central	+	I	/	/
9	Quadriceps femoris	+ ±	–	+	20%	Central	–	I	/	/
10	/	/	/	/	/	/	/	/	/	/
11	/	/	/	/	/	/	/	/	/	/
12	Quadriceps femoris	±	–	–	40%	Central	–	Normal	+	–
13	/	/	/		/	/	/	/	/	/
14	/	/	/		/	/	/	/	/	/
15	Quadriceps femoris	+ + / +	–	–	20%	Central	–	Normal	/	/

" +  +  + " indicates characteristic/strong, where " + " indicates mild, and " +  + " indicates moderate."−" indicates none. "/" means no electron microscopy done

### Genetic analysis results

All 15 patients underwent genetic analysis and were identified to carry variants in one of three NM related genes including *NEB* (n = 11, 73.33%), *ACTA1* (n = 1, 6.67%), *TPM3* (n = 3, 20.00%). In total, 9 mutations were harbored in *NEB* genes of which 8 (88.89%) in exon and 1(11.11%) in the intron. The splicing mutation in the intron region carried by 10 patients were all c.21522 + 3A > G (Fig. 2). The other 8 mutations were in exon and included frameshift mutation c.1623delT in 3 (37.50%) patients, nonsense mutation c.17611C > T in 3(37.50%) patients, nonsense mutation c.4417C > T in 2 (25.00%) patients, which both had been reported previously. The remaining 6 mutations were novel, including c.17611C > T in 3 patients, and c.2549delA, c.3520G > A, c.21065dupA, c.20943G > A, c.192G > A, each in 1 patient respectively. We identified 2 novel mutations in 3 patients with *TPM3*, among which one was splicing mutation c.642 + 5G > A in the intron, the other was missense mutation c.*25G > A in exon. A de novo missense mutation c.745G > A in exon was identified in 1 patient with *ACTA1*.

## Discussion

The clinical phenotype of NM has great heterogeneity. The proximal limb muscles are usually weak at onset, and the clinical symptoms progress slowly. Their enrollment was based on the following inclusion criteria [1, 2, 19]: (1) they clinically presented with muscle weakness,amyotrophy,hypotonia,delayed motor milestones and dysmorphic features; (2) muscle biopsy showed the presence of rods in muscle fibers after light and electron microscopy analysis, and no other characteristic pathological findings; (3) they did not have other diseases, such as muscular dystrophy, congenital myasthenic syndromes, or immuno-/metabolic myopathy. However, in recent years, some studies had reported the onset of distal muscle weakness, especially in NM with NEB gene mutation [20–22]. In our study, 2 patients with NEB gene mutation (Case1 and Case10) all developed distal limb weakness, manifested as dorsiextension weakness and inability to walk fast, and proximal limb muscle weakness gradually developed as the disease progressed, which was consistent with the report of Vilma-Lotta et al. [21]. Different from previous literature, this group of patients mainly had weakness in both lower limbs, and muscle weakness in both upper limbs gradually appeared 20 years after onset, and the clinical course progressed very slowly. And 10 out of 15 patients (66.7%) had arch palate and elongated faces, which indicates that the two are important features in NM patients.

The rod aggregates observed in muscle fibers in muscle fibers in muscle biopsy is an important pathological feature for the diagnosis of NM. MGT staining is the main staining method for the diagnosis of NM, and rod-shaped granular material stained purple and blue can be found in muscle fibers, which is the characteristic pathological change of NM. The rods mainly gather in subsarcolemmal and the center of muscle fibers, but can also be observed in normal and atrophic muscle fibers. The percentage of fibers containing rods was calculated more than 50% in 5/9 cases, and greater than 90% in 3/9 cases. The remaining four cases were 40%, 30%, 30% and 20%, respectively. None of the characteristic pathological changes of other muscle diseases were observed in any patients. The number of rods increased with age but there was no obvious correlation with the severity of disease. HE staining showed that dark red eosinophilic deposits mostly located under the sarcolemma could be seen in fibers of some patients, which were consistent with the positions of rods seen in fibers of MGT staining. ATP staining revealed that 7 out of 9 cases had abnormal distribution of fibers type, of which type I predominance in 5 cases and type II predominance in 2 cases. It also confirmed that most NM patients had aberrant distribution of fibers. However, the histology of patients with NM caused by *TPM3* and *ACTA1* indicated that type I and II fibers still presented mosaic distribution, and no obvious groupings were found, which suggested that NM caused by *NEB* gene was more prone to aberrant distribution and groupings of type I and II fibers. The mechanism of this phenomenon was still unclear. Electron microscopic analysis of 6 patients showed that rods mostly located near the Z band were observed in the myofibrils or subsarcolemmal in 5 cases. And this might be explained by the main component of the rods was α-actinin, which was the same as that of the Z line.

Until now, 14 genes have been reported to be related with NM. Among them, *NEB* is the most frequent and may account for up to 50% of cases [23]. *NEB* gene was identified in 11 out of 15 patients in our cohort, accounting for about 73%, which was consistent with previous reports [24]. The autosomal recessive mutation was most common in *NEB*, and it could also be sporadic, and autosomal dominant mutations was rare but have been reported [22]. The 11 patients with *NEB* mutation came from 7 families respectively, of which 6 patients were from two families. However, the 6 patients were all from the same generation, and the rest of their families were all healthy, which is consistent with autosomal recessive inheritance. The 11 *NEB* related NM patients were found to have compound heterozygous mutation. A total of 9 mutation sites were identified, including 8 mutations in exon and 1 mutation in the intron. In addition, the splicing mutation c.21522 + 3A > G in *NEB*, carried by 10 patients, was predicted to affect the splicing function of mRNA and lead to changes in amino acids, and it was classified as a pathogenic mutation [25]. At present, the mutation c.21522 + 3A > G has been reported in China and South Korea, which can cause childhood-onset NM [26, 27]. Yin et al. [28] found that c. 21522 + 3A > G was the most common mutation of *NEB* gene in a group of NM cases reported, which was consistent with our study. Wang et al. [29] reported in a study that the *NEB* gene mutation C.21417 + 3A > G firstly reported in South Korea may be a mutational hotspot in East Asian populations. Based on the results of this study, we consider that c.21522 + 3A > G may be another mutational hotspot of *NEB* gene in Chinese and East Asian populations.

The most common types of mutations observed in the *NEB* gene were missense, frameshift, and nonsense mutations, while the rarest types were the copy number and synonymous mutations [30]. There were 8 mutation sites in exons, including 3 frameshift mutations, 2 missense mutations, 1 nonsense mutations, 2 synonymous mutations, and no copy number variation was found. Except for one nonsense mutation c.4417C > T and one frameshift mutation c.1623delT, which both had been reported before [29, 30]. The remaining six mutation sites, c.17611C > T, c.2549delA, c.3520G > A, c.21065dupA, c.20943G > A and c.192G > A, have not been reported, so they should be novel variants of *NEB* gene.

*ACTA1* gene mutation is the second most common one that causes NM. The majority of reported mutations are autosomal dominant and rare can be autosomal recessive [23]. The clinical manifestations of nemaline myopathy caused by *ACTA1* gene mutations is often severe, more rarely, mutations in *ACTA1* may cause the intermediate, mild, or typical Forms of nemaline myopathy [28, 31, 32]. The clinical subtype of the case in our cohort is mild, which is a rare one. A single heterozygous missense mutation, c.745G > A, in exon region of *ACTA1* was harbored, which could result in disrupted protein structure and finally impaired muscle contraction. The patients showed no remarkable family history; hence, we also speculated that these were de novo mutations.

Mutations in *TPM3* account for only a small percentage of patients with NM, which have been associated with both autosomal dominant NM, and rarely with autosomal recessive NM [33]. We identified 2 novel heterozygous mutations in 3 patients with *TPM3* mutations that came from the same family. One was c.642 + 5G > A, a splice mutation in the intron region, which was predicted to affect the splicing function of mRNA and lead to changes in amino acids. Another was c.*25G > A, a missense mutation in the exon region, which caused amino acid alterations. The two mutations in *TPM3* could be rated as meaningless mutations (VUS) according to the ACMG guidelines.

## Conclusions

In conclusion, the clinical phenotype of NM is highly heterogeneous, and the age of onset in NM is mostly in childhood, with proximal limb muscle weakness as the main clinical manifestation. Some patients first present with distal lower extremity weakness that gradually progress to involve the proximal muscle. The rod aggregates observed in muscle biopsy is an important pathological feature ©for the diagnosis of NM. *NEB* is the most common NM causative gene in the present cohort of Chinese patients, and splicing mutation c.21522+3A>G in *NEB* is the most frequently found mutation in our patients. This is a novel finding and may indicate a new hotspot mutation in Chinese NM patients.

## Data Availability

The datasets used and/or analyzed during the current study are available from the corresponding author on reasonable request.
